# A Comparison of Hypotension, Bradycardia, and Hypoxia Incidence between the Use of Remimazolam and Other Sedative Agents during Colonoscopy Procedures: A Systematic Review and Meta-Analysis

**DOI:** 10.3390/jcm13154352

**Published:** 2024-07-25

**Authors:** Chia-Hao Ho, Cheng-Ying Chang, Cheng-Wei Lu

**Affiliations:** 1 Department of Anesthesiology, Far Eastern Memorial Hospital, New Taipei City 22060, Taiwan; tad820423@gmail.com (C.-H.H.); chengying0522@gmail.com (C.-Y.C.); 2 Department of Mechanical Engineering, Yuan Ze University, Taoyuan 32003, Taiwan

**Keywords:** remimazolam, safety, hemodynamics

## Abstract

**(1) Background:** Remimazolam is a newly developed sedative agent. The results of previous meta-analyses highlight the strengths of remimazolam for use during colonoscopy procedures. The primary aim of the present study was to investigate whether, in patients undergoing colonoscopy procedures (P), the use of remimazolam (I) compared with other sedative agents (C) could lead to a greater incidence of hypotension, bradycardia, and hypoxia (O). **(2) Methods:** In the following study, we conducted an extensive literature search using two electronic databases. We included all randomized control trials, which involved a comparison of the hemodynamic changes in remimazolam versus a placebo and other sedative agents during colonoscopy procedures. Data extraction, data synthesis, and the assessment of risk of bias were performed by the authors. **(3) Results:** A total of seven articles met our inclusion criteria. The combined analysis of the selected studies revealed no statistically significant difference in hypotension, bradycardia, or hypoxia incidence when comparing remimazolam and the control group. However, in comparison with the group administered propofol, the pooled data of the selected studies revealed statistically significant differences in the incidence of both hypotension and bradycardia but not hypoxia. **(4) Conclusions:** Our findings indicate that there is no significant difference in hypotension, bradycardia, and hypoxia incidence when comparing remimazolam and other agents. Nevertheless, when comparing the remimazolam and propofol groups, the results demonstrated statistically significant differences in the incidence of both hypotension and bradycardia but not hypoxia.

## 1. Introduction

Remimazolam is a newly developed benzodiazepine analog with an ester modification. Its mechanism of action involves targeting the GABA type A (GABA-A) chloride ionophore receptor complex, similar to its precursor midazolam. This innovative drug is a fusion of midazolam and remifentanil, featuring an incorporated carboxylic ester linkage designed to facilitate metabolic functions [1]. The results of previous reviews have revealed that remimazolam demonstrates highly effective procedural sedation, outperforming midazolam in several key areas. Studies have shown that remimazolam not only achieves higher success rates in sedation but also provides a more favorable recovery profile for patients. However, further comparisons with propofol are necessary [2,3].

Hypotension frequently occurs during colonoscopy procedures under propofol sedation, presenting a concern due to its potential adverse impact on patient safety and outcomes [4]. The severity and duration of hypotension observed during propofol sedation for colonoscopy procedures are comparable to those observed in surgical settings. The above highlights the significance of closely monitoring blood pressure levels and implementing appropriate measures to mitigate the risks associated with hypotension during colonoscopy procedures under propofol sedation. Implementing an early warning system (EWS) alongside a hemodynamic algorithm may lead to a decreased incidence of intraoperative hypotension in addition to lower levels of neuronal specific enolase (NSE) and oxidative stress [5]. Data from previous studies also show that dynamic indices are superior to static indices for predicting fluid responsiveness in critically ill patients during medical procedures [6]. These monitoring methods have the potential to reduce postoperative or postprocedural morbidity and mortality.

In contrast, the authors of a previous study stated that sedation is commonly employed to alleviate anxiety associated with endoscopic procedures, mitigate procedural risks, and enhance patient comfort and satisfaction. Administering propofol under the direction of a gastroenterologist is a safe and effective option compared to sedation using opioids and benzodiazepines. Prior to implementing a propofol sedation program, both physicians and nursing staff require specialized training [7,8].

In a previous meta-analysis, the results of a comparison between the use of remimazolam and propofol for general anesthesia revealed several advantages favoring remimazolam [9]. Notably, the administration of remimazolam resulted in a reduction in the incidence of hypotension, hypoxemia, nausea and vomiting, dizziness, and injection site pain when compared with propofol. Additionally, patients administered remimazolam exhibited more stable mean arterial pressure (MAP) both before and after intubation, further bolstering its safety profile as a sedative agent for anesthesia induction. In addition, the strengths of remimazolam in patients undergoing colonoscopy procedures have been highlighted in comparison to other agents; moreover, researchers in countries worldwide, including those in Europe and the United States, are conducting trials on remimazolam for use in procedural sedation [10]. Nonetheless, specific aspects of this medication require meticulous scrutiny, such as its safety profile, before it can be widely embraced and adopted. The primary aim of the following study was to investigate whether, in patients undergoing colonoscopy procedures (P), the use of remimazolam (I) compared with the use of a placebo or other sedative agents (C) could lead to a greater incidence of hypotension, bradycardia, and hypoxia (O).

## 2. Materials and Methods

The study presented herein adhered to the guidelines outlined in the Cochrane Handbook for Systematic Review of Interventions and followed the Preferred Reporting Items for Systematic Reviews and Meta-Analysis (PRISMA) guidelines regarding its composition [11,12]. This study’s protocol was registered in the International Prospective Register of Systematic Reviews (PROSPERO) in 2023 (registration number: CRD42023465760).

### 2.1. Search Strategy

We conducted an extensive literature search by searching two electronic databases, namely PubMed and the Cochrane Central Register of Controlled Trials. Our search covered a substantial period, spanning from 1 January 1980 to 21 September 2023, ensuring a comprehensive review of the relevant literature over a period of more than four decades. To facilitate this search, we employed specific search strings, namely “remimazolam” and “colonoscopy”, and utilized MeSH (Medical Subject Headings) term searching to enhance the precision and relevance of our results.

Our approach did not impose any language restrictions, allowing for us to capture a diverse range of studies from various linguistic backgrounds. This inclusivity aimed to provide a broad perspective of the available evidence. However, we established criteria to maintain the quality and reliability of our findings. Specifically, we excluded unpublished studies and those without full-text availability, as these limitations could hinder thorough review and analysis. By setting these parameters, we aimed to ensure that the studies included in our review were accessible and provided sufficient data for a robust evaluation of the topic in question.

### 2.2. Study Selection

#### 2.2.1. Inclusion Criteria

We included all randomized controlled trials (RCTs) that compared the hemodynamic changes associated with the use of remimazolam to those observed with other commonly used sedative agents during colonoscopy procedures. Specifically, the researchers involved in these trials examined and contrasted remimazolam with a placebo and other sedative agents, such as midazolam, propofol, and etomidate, for use in colonoscopy procedures.

#### 2.2.2. Exclusion Criteria

We excluded several types of studies to maintain the focus and quality of our review. Specifically, unpublished studies, observational studies, reviews, and case reports were not included. Additionally, studies published only in abstract form were excluded, as such studies often lack the detailed data necessary for thorough analysis. We also excluded articles investigating other procedural interventions, such as gastroscopy and bronchoscopy, to ensure that our review remained concentrated solely on colonoscopy procedures and the associated hemodynamic effects of different sedative agents.

### 2.3. Outcomes

#### 2.3.1. Primary Outcomes

The primary outcomes were the incidence of hypotension, bradycardia, and hypoxia between the group administered remimazolam and the group administered other sedative agents.

#### 2.3.2. Secondary Outcomes

The secondary outcomes were the incidence of hypotension, bradycardia, and hypoxia between the group administered remimazolam and the group administered propofol.

### 2.4. Data Extraction

Two authors (Ho C.H. and Chang C.Y.) assessed every article independently, evaluated whether it met the inclusion criteria, and used standardized data collection forms to perform data extraction. We extracted data comprising continuous variables, including the mean and standard deviation (SD), and dichotomous data, including the number of events that occurred and the sample size. If there were any discrepancies in our selections and the data extraction results, consensus would be sought by consulting the third author (Lu C.W.).

### 2.5. Data Synthesis

If more than 50% of the studies meticulously listed outcome data with consistent definitions and units, the extracted data from eligible articles were synthesized appropriately. Meta-analyses were performed using RevMan 5.4 software, The Nordic Cochrane Centre, Copenhagen, Denmark (https://training.cochrane.org/online-learning/core-software-cochrane-reviews/revman/revman-5-download, accessed on accessed on 1 October 2023).

We utilized a random-effects model to account for clinical and methodological heterogeneity across studies. Statistical heterogeneity was assessed using I2 statistics and the Q test. I2 values ranging from 30% to 60% were categorized as moderate heterogeneity, while values between 50% and 90% indicated substantial heterogeneity. Weighted mean differences (WMDs) of mean values and standard deviations (SDs) were calculated using the inverse variance method exclusively for outcomes in our analysis. Statistical significance was defined as a *p*-value < 0.05.

### 2.6. Risk of Bias

Two review authors (Ho C.H. and Chang C.Y.) independently evaluated the risk of bias in the selected eligible studies using the “Risk of Bias” assessment tool from the Cochrane Handbook. They created a summary figure of the risk of bias using Review Manager (RevMan 5.4.1). For the overall risk-of-bias assessment, the selected studies were categorized as follows: Those deemed to have a low risk of bias across all domains were classified as “low risk”; studies raising some concerns in more than one domain without any high risk of bias were classified as “Some concerns”; and studies assessed to have a high risk of bias in more than one domain were classified as “high risk”.

### 2.7. Assessments of Confidence

We evaluated the quality of evidence for the outcomes under investigation using the Grading of Recommendations Assessment, Development, and Evaluation (GRADE) system. This assessment involved scrutinizing study limitations, consistency of effects, imprecision, indirectness, and publication bias within our reviews [13]. Following this evaluation, we generated a GRADE evidence profile table utilizing GRADEpro software. (https://www.gradepro.org/, accessed on 1 October 2023) to categorize all outcomes as very low, low, moderate, or high quality.

## 3. Results

### 3.1. Search Results

**Figure 1:**
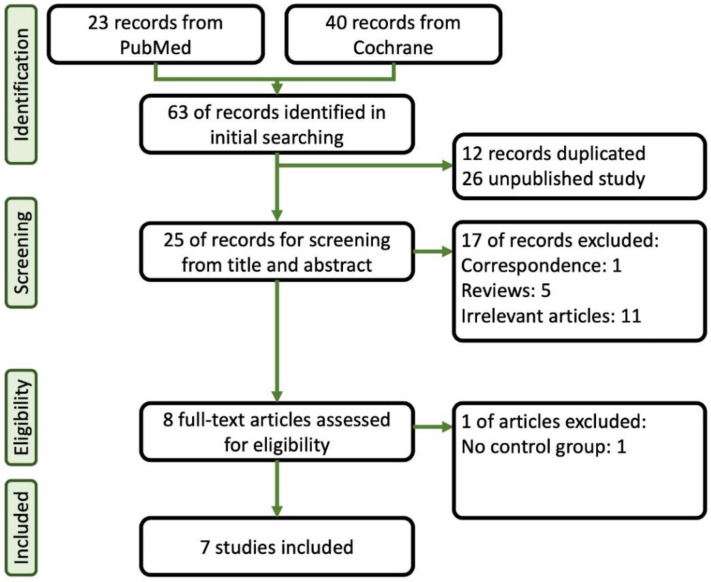
Flowchart of the systematic review followed by the search strategy.

A total of 23 papers were identified through a search of PubMed, and 40 papers were sourced from Cochrane. Initially, duplicate and unpublished studies were excluded, leaving 25 studies for further assessment. These studies underwent careful screening based on their titles and abstracts. Among them, seven articles that met our inclusion criteria and provided sufficient statistical data were ultimately included in the final analysis [14,15,16,17,18,19,20]. Figure 1 provides a summary of the flowchart of the database search process. Table 1 provides a summary of the characteristics of the selected studies, which include the American Society of Anesthesiologists (ASA) Classification, case numbers, comparison agents, age, and body mass index (BMI). As shown in Table 2, we summarized the doses of induction and maintenance of remimazolam, other sedative agents, and opioids used in these studies during colonoscopy procedures. Additionally, in Table 3, we provide the definitions of hypoxia, hypotension, and bradycardia as described in each article.

### 3.2. Primary Outcomes

**Figure 2:**
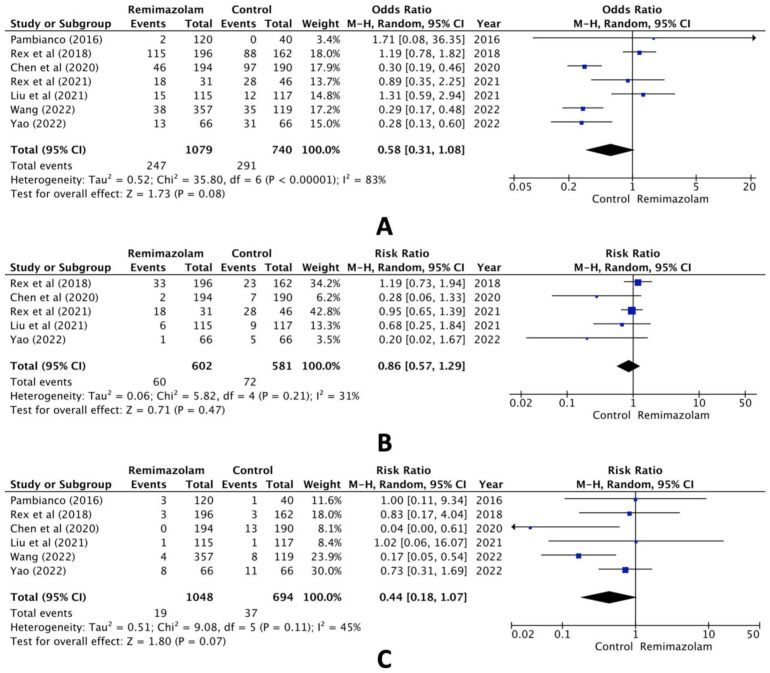
Forest plot of the incidence of (**A**) hypotension, (**B**) bradycardia, and (**C**) hypoxia (remimazolam group versus the control group) [14,15,16,17,18,19,20].

When comparing the group administered remimazolam and the group administered other sedative agents, the combined analysis of the seven selected studies revealed no statistically significant difference in hypotension (RR, 0.58; 95% CI, 0.31 to 1.08; n = 1819; *p* = 0.08; I^2^ = 83%, Figure 2A). Pooled data also showed no statistically significant difference in the incidence of bradycardia (RR, 0.86; 95% CI, 0.57 to 1.29; n = 1183; *p* = 0.47; I^2^ = 31%, Figure 2B) or hypoxia (RR, 0.44; 95% CI, 0.18 to 1.07; n = 1742; *p* = 0.07; I^2^ = 45%, Figure 2C).

### 3.3. Secondary Outcomes

**Figure 3:**
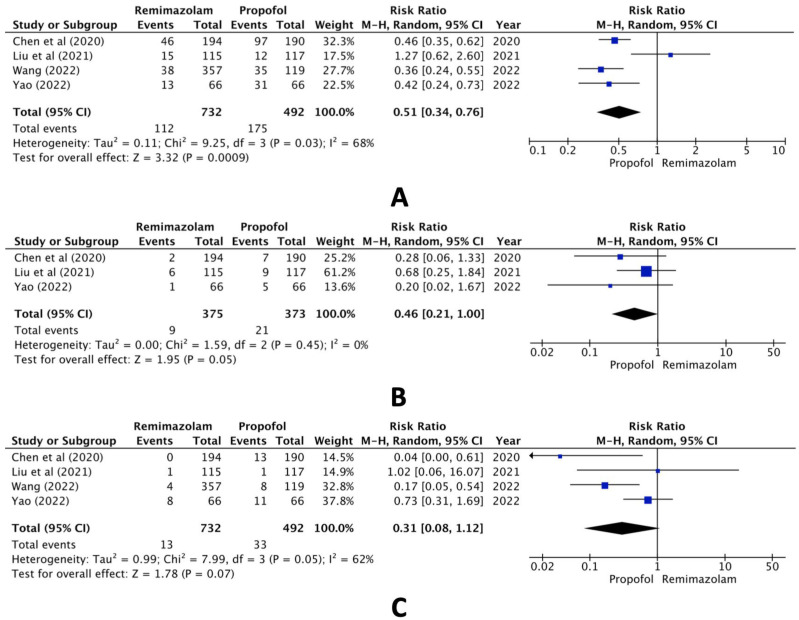
Forest plot of the incidence of (**A**) hypotension, (**B**) bradycardia, and (**C**) hypoxia (remimazolam group versus the propofol group) [16,18,19,20].

In the comparison between the remimazolam and propofol groups, however, pooled data of the selected studies revealed statistically significant differences both in hypotension (RR, 0.51; 95% CI, 0.34 to 0.76; n = 1224; *p* = 0.03; I2 = 68%, Figure 3A) and bradycardia (RR, 0.46; 95% CI, 0.21 to 1.00; n = 749; *p* = 0.05; I2 = 0%, Figure 3B) but not hypoxia (RR, 0.31; 95% CI, 0.08 to 1.12; n = 1224; *p* = 0.07; I2 = 62%, Figure 3C).

### 3.4. Risk of Bias

**Figure 4:**
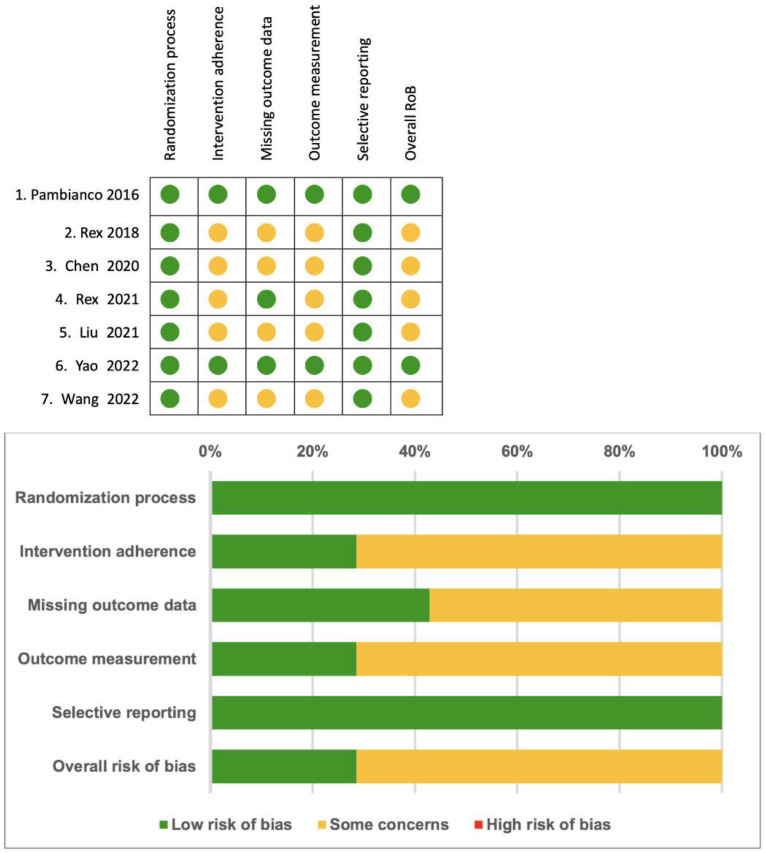
Summary of the risk of bias [14,15,16,17,18,19,20].

In Figure 4, a summary of the risk of bias is provided. The selected trials were classified as having at least a moderate risk of bias overall, primarily due to the inability of operators to be blinded to the type of sedative agents used in each trial, and outcome measurement.

### 3.5. Assessments of Confidence

We assessed the quality of evidence using the GRADE assessment [13], with Table S1 providing a concise overview of both the quality of evidence and the findings. Owing to heterogeneity, the differences in the definition of hypotension, bradycardia, and hypoxia and the inability to blind the participants, the majority of outcomes were classified as low quality, highlighting a limitation of our study.

## 4. Discussion

The objective of this study was to assess the safety of the sedative agent remimazolam compared to other sedative agents in patients undergoing colonoscopy procedures, and our findings indicated that there were no significant differences in the incidence of hypotension, bradycardia, and hypoxia in the comparison between the use of remimazolam and other agents in the articles examined. Nevertheless, when comparing the remimazolam and propofol groups, the results demonstrate statistically significant differences in both hypotension and bradycardia incidence. A meta-analysis, investigating safety and efficacy in endoscopy procedures, showed remimazolam to be a secure and efficient sedative option for patients undergoing endoscopic procedures [3]. This conclusion is similar to that of our study. Moreover, hypotension frequently occurs during propofol sedation for colonoscopy procedures, reaching levels of severity and duration that can pose risks similar to those observed in surgical patients [4]. Therefore, when comparing remimazolam and propofol, we can observe significant differences.

The higher efficacy of remimazolam may be attributed to its distinct mechanism of action. Remimazolam has a heightened affinity and selectivity for the benzodiazepine site on GABA receptors, resulting in more potent sedative effects. Additionally, unlike midazolam’s metabolite α-hydromidazolam, the metabolite of remimazolam exhibits a reduced affinity for benzodiazepine receptors, further contributing to its enhanced efficacy and favorable pharmacokinetic profile. These characteristics make remimazolam particularly effective for procedural sedation [21,22,23]. As a result, its use in endoscopic examinations is gaining increasing attention. A number of researchers are currently focusing on evaluating its safety and efficacy in such procedures, aiming to provide robust evidence highlighting its clinical advantages and optimize patient outcomes in endoscopic settings.

The results of previous comprehensive meta-analyses indicate that remimazolam exhibits potential superiority over midazolam as a preferred option for procedural sedation [3,24]. These findings highlight several advantages of remimazolam, including but not limited to its efficacy, safety profile, and patient tolerability. However, the study included three procedures, including endoscopy, colonoscopy, and bronchoscopy, and the researchers compared groups administered either remimazolam or midazolam. In comparison with previous studies, our research only included studies related to colonoscopy procedures and compared the groups administered either remimazolam, other sedative agents, or propofol. The above factors indicate that our study provides more convincing evidence for improvement in patients undergoing colonoscopy procedures under general conditions.

Following studies on endoscopy procedures that have identified the benefits of remimazolam, numerous studies have been conducted on remimazolam and its association with general anesthesia. When compared to propofol, the use of remimazolam for general anesthesia decreased the incidence of hypotension, hypoxemia, nausea and vomiting, dizziness, and injection site pain [9,25]. Moreover, remimazolam offers advantages over propofol, notably in terms of safety, characterized by reduced hemodynamic instability and respiratory depression, the absence of injection pain, and the availability of a known reversible agent, flumazenil [26,27]. These combined advantages underscore remimazolam’s potential as a preferred sedative agent in various clinical settings, offering both efficacy and enhanced safety for patients undergoing procedural sedation.

When using sedative drugs, it is also necessary to consider the potential for addiction. The addictive nature of propofol has become a medical issue that cannot be ignored [28]. Remimazolam, as a new type of benzodiazepine, similarly necessitates consideration of the potential for remimazolam-induced addiction. Fortunately, the results of a recent study indicate that remimazolam has a very low potential for intravenous abuse [29]. Additionally, the high price of remimazolam may lead to concerns about its use. However, Pedersen and colleagues found that the use of remimazolam for procedural sedation in patients undergoing colonoscopies and bronchoscopies is economically beneficial, with cost savings when compared to midazolam and propofol, due to lower total costs and reduced time spent in preparation and recovery phases. Remimazolam offers rapid onset and recovery, contributing to its cost-effectiveness and operational efficiency in procedural sedation [30].

The findings of previous meta-analyses suggest that the use of remimazolam enables better maintenance of hemodynamic stability compared to sevoflurane when used for general anesthesia. This advantage is particularly significant given the complex physiological challenges associated with certain surgical procedures. For instance, during robotic gastrectomy, patients often experience hypotension due to the combined effects of CO_2_ pneumoperitoneum, which increases intra-abdominal pressure, and the head-up position they must assume, which can alter venous return and cardiac output. The ability of remimazolam to maintain more stable blood pressure and heart rate under these conditions implies that it could be especially beneficial for patients at a higher risk of hemodynamic instability. Consequently, incorporating remimazolam into anesthesia protocols for such surgeries may improve patient outcomes by reducing the incidence and severity of intraoperative hypotensive episodes, thereby enhancing overall surgical safety and recovery [31].

Flumazenil, the antagonist of benzodiazepines, is essential in reversing the sedative effects of benzodiazepines, offering a crucial safety measure in clinical situations requiring rapid recovery from sedation. Based on current evidence, the combination of remimazolam and flumazenil accelerates recovery from general anesthesia and reduces the risk of respiratory depression compared to propofol. However, the higher risk of re-sedation associated with this combination should be carefully considered in clinical practice [32]. However, there is a lack of evidence regarding the use of the combination of remimazolam and flumazenil in patients undergoing endoscopy and colonoscopy procedures.

We found two similar meta-analyses during our literature search. The results of Ahmer et al.’s study show that remimazolam demonstrates superior safety compared to propofol when used in gastrointestinal endoscopy and colonoscopy procedures among elderly patients [33]. The strength of our study lies in its focus on colonoscopy procedures and the comparison of remimazolam with other sedative agents across all age groups. The results of Ul-Haque et al.’s study highlight the advantages of remimazolam over midazolam [10]. However, given the variety of agents available for colonoscopy procedures, our analysis could be further strengthened by including comparisons with other sedative agents.

In addition to the aforementioned limitations, it is important to acknowledge several other constraints of our analysis. Firstly, the heterogeneity among study protocols, strategies, and endpoints may have introduced variability and potential bias in the interpretation of both primary and secondary outcomes. This variability complicates direct comparisons and may affect the overall conclusions drawn from the data. Secondly, the exclusive enrollment of adult participants in the studies reviewed limits the generalizability of our findings to pediatric populations. This exclusion criterion underscores the need for separate investigations focusing on children and adolescents to determine the safety and efficacy of remimazolam in these age groups. Pediatric patients often have different physiological responses to medication, and dedicated research is essential to address these differences. Thirdly, the variability in ASA classification among the studies in the included articles, predominantly clustering within ASA 1-2, raises concerns about the applicability of remimazolam in higher-risk patient populations. Further research addressing these limitations is warranted to ensure a comprehensive understanding of remimazolam’s efficacy and safety across diverse patient demographics and clinical scenarios.

We conducted a trial sequential analysis, with the results indicating that remimazolam may have a favorable effect compared to other sedative agents and placebos; however, this evidence is not yet conclusive. Future additional trials may be required in order to acquire a sufficient amount of information and may potentially have to cross the trial sequential monitoring boundaries for a definitive conclusion to be made.

## 5. Conclusions

Remimazolam holds significant promise as a future alternative to propofol for sedation induction in gastrointestinal endoscopy procedures. Its potential to enhance patient safety, reduce recovery times, and offer a more predictable sedation profile positions remimazolam as a critical advancement in the field. As research and clinical experience grow, remimazolam is poised to become an essential tool for improving procedural outcomes and patient experiences in gastrointestinal endoscopy.

In conclusion, the results of our analysis suggest that the use of remimazolam in patients undergoing colonoscopy procedures does not lead to greater incidence of hypotension, bradycardia, and hypoxia compared to the use of other sedative agents. When comparing the remimazolam and propofol groups, the results demonstrate a greater incidence of both hypotension and bradycardia. However, for high-risk populations and other procedures requiring patient sedation, further research is required for validation of the above results.

## Figures and Tables

**Table 1:** The summary of the characteristics of selected studies.

No	Country	Year	Author	ASA ^1^	Case (R ^2^/C ^3^)	Comparison	Age (Years)	BMI ^4^ (kg/m^2^)
1	US	2016	Pambianco [14]	1–2	160 (120/40)	Midazolam	Overall mean 55	Overall mean 27
2	US	2018	Rex [15]	1–3	458 (296/162)	Placebo n = 60, Midazolam n = 102	Remimazolam 54.4 ± 10.12 Placebo 56.0 ± 9.51 Midazolam 55.6 ± 10.15	Remimazolam 28.9 ± 4.72 Placebo 30.0 ± 5.31 Midazolam 28.8 ± 4.75
3	China	2020	Chen [16]	1–2	384 (194/190)	Propofol	Remimazolam 44.47 ± 11.67 Propofol 44.43 ± 11.37	Remimazolam 23.19 ± 2.92 Propofol 23.21 ± 2.84
4	US	2021	Rex [17]	3–4	77 (31/46)	Placebo n = 16, Midazolam n = 30	Remimazolam 63.1 ± 8.6 Placebo 63.0 ± 8.37 Midazolam 61.5 ± 10.60	Remimazolam 30.9 ± 8.24 Placebo 30.8 ± 5.53 Midazolam 30.8 ± 6.75
5	China	2021	Liu [18]	1–2	232 (115/117)	Etomidate-Propofol	Remimazolam 68.87 ± 2.58 Etomidate-Propofol 69.12 ± 2.75	Remimazolam 25.35 ± 2.07 Etomidate-Propofol 24.75 ± 2.16
6	China	2022	Wang [19]	1–3	476 (357/119)	Propofol	Remimazolam 44.3 [33.0–54.0] Propofol 46.4 [37.5–56.0]	Remimazolam 22.82 [20.70–24.90] Propofol 22.87 [21.10–24.60]
7	China	2022	Yao [20]	1–2	132 (66/66)	Propofol	Remimazolam 49 [41–56] Propofol 48 [39–56]	Remimazolam 22.4 [19.8–24.6] Propofol 22.0 [20.1–25.3]

^1^ ASA = American Society of Anesthesiologists Classification. ^2^ R = remimazolam. ^3^ C = comparison. ^4^ BMI = body mass index. Data are presented as mean ± SD, median [IQR].

**Table 2:** The summary of doses of induction and maintenance of remimazolam, other sedative agents, and opioids in colonoscopy.

No	Country	Year	Author	Dose of Remimazolam (Induction/Maintenance)	Dose of other Sedative Agents (Induction/Maintenance)	Opioids Use (Induction/Supplemental Dose)
1	US	2016	Pambianco [14]	8.0 mg/3.0 mg, 7.0 mg/2.0 mg, or 5.0 mg/3.0 mg	2.5 mg/1 mg	100 mcg/25 mcg of fentanyl
2	US	2018	Rex [15]	5.0 mg/2.5 mg	1.75 mg/1.0 mg for <60 years old patients 1.0 mg/0.5 mg for >60 years old patients	80% patients 75 mcg/25 mcg of fentanyl 20% patients 50 mcg/25 mcg of fentanyl
3	China	2020	Chen [16]	5.0 mg/2.5 mg	1.5 mg/kg/0.5 mg/kg	^2^ Remimazolam 62.99 ± 10.81 of fentanyl ^2^ Propofol 63.64 ± 11.19 of fentanyl
4	US	2021	Rex [17]	2.5-5.0 mg/1.25-2.5 mg	1 mg/0.5 mg	50 mcg/25 mcg of fentanyl
5	China	2021	Liu [18]	0.15 mg/kg/0.075 mg/kg	E-P ^1^ 0.1 mL/kg/0.05 mL/kg	0.5 mcg/kg/0.5 mcg/kg of fentanyl
6	China	2022	Wang [19]	7 mg/2.5 mg	1.5 mg/kg/0.5 mg/kg	50 mcg of fentanyl
7	China	2022	Yao [20]	0.2 mg/kg/6 mg	1 mg/kg/30 mg	5 mcg of sufentanil

^1^ E-P = Etomidate-propofol, 10 mL/20 mg etomidate plus 10 mL/100 mg propofol. ^2^ Data are presented as mean ± SD.

**Table 3:** The summary of the definitions of outcomes in each study, such as hypoxia, hypotension, and bradycardia.

No	Country	Year	Author	Hypoxia	Hypotension	Bradycardia
1	US	2016	Pambianco [14]	Oxygen saturation < 90%	SBP ^1^ < 80 mmHg	Not mentioned in the article
2	US	2018	Rex [15]	Oxygen saturation < 90% for ≥1 min or any drop necessitating medical intervention	SBP ≤ 80 mmHg or DBP ^2^ ≤ 40 mm Hg, or a fall in SBP or DBP of 20% or more below baseline or necessitating medical intervention	<40 bpm ^4^ or drop in heart rate of 20% > 30 s
3	China	2020	Chen [16]	Oxygen saturation < 90%	The reduction of SBP ≥ 20% (compared to baseline SBP before sedation) or decreased to ≤80 mmHg	Not mentioned in the article
4	US	2021	Rex [17]	Oxygen saturation < 90% for ≥1 min, or any drop necessitating medical intervention	SBP ≤ 80 mmHg or DBP ^2^ ≤ 40 mm Hg, or a fall in SBP or DBP of 20% or more below baseline or necessitating medical intervention	<40 bpm ^4^ or drop in heart rate of 20% > 30 s
5	China	2021	Liu [18]	Oxygen saturation < 90%	SBP < 90 mmHg, DBP < 50 mmHg, or a MAP ^3^ decrease of 20% or more below baseline	<50 bpm or a decrease in heart rate of 20% or more from baseline
6	China	2022	Wang [19]	Oxygen saturation < 90%	SBP ≤ 80 mmHg	Not mentioned in the article
7	China	2022	Yao [20]	Oxygen saturation < 92%	Decrease in MAP at least 20% from the baseline value	Heart rate less than 50 bpm

^1^ SBP = systolic blood pressure. ^2^ DBP = diastolic blood pressure. ^3^ MAP = mean blood pressure. ^4^ bpm = beats per minute.

## Supplementary Materials

The following supporting information can be downloaded at: https://www.mdpi.com/article/10.3390/jcm13154352/s1. PRISMA_2020_checklist. Table S1: GRADE evidence profiles of outcomes.
