# Supplementary material for: A Comparison of Hypotension, Bradycardia, and Hypoxia Incidence between the Use of Remimazolam and Other Sedative Agents during Colonoscopy Procedures: A Systematic Review and Meta-Analysis
Source: J Clin Med. 2024 Jul 25;13(15):4352. doi: 10.3390/jcm13154352 (PMC11313025; doi:10.3390/jcm13154352)
Supplement: Supplementary file 1 [file jcm-13-04352-s001.zip › Table S1.pdf]

Table S1. GRADE evidence profiles of outcomes

Certainty assessment							Summary of findings				
							Study event rates (%)			Anticipated absolute effects	
Participants (studies) Follow-up	Risk of bias	Inconsistency	Indirectness	Imprecision	Publication bias	Overall certainty of evidence	With Other sedative agents	With Remimazolam	Relative effect (95% CI)	Risk with Other sedative agents	Risk difference with Remimazolam
**Hypotension**											
1819 (7 RCTs)	serious	serious	not serious	not serious	none	⨁⨁◯◯ Low	291/740 (39.3%)	247/1079 (22.9%)	**OR 0.581** (0.310 to 1.080)	393 per 1,000	**120 fewer per 1,000** (from 226 fewer to 19 more)
**Bradycardia**											
1183 (5 RCTs)	serious	serious	not serious	not serious	none	⨁⨁◯◯ Low	72/581 (12.4%)	60/602 (10.0%)	**OR 0.86** (0.57 to 1.29)	124 per 1,000	**15 fewer per 1,000** (from 49 fewer to 30 more)
**Hypoxia**											
1742 (6 RCTs)	serious	serious	not serious	not serious	none	⨁⨁◯◯ Low	37/694 (5.3%)	19/1048 (1.8%)	**OR 0.44** (0.18 to 1.07)	53 per 1,000	**29 fewer per 1,000** (from 43 fewer to 4 more)

CI: confidence interval; **OR:** odds ratio
